# Recent progress and perspectives on prostate cancer biomarkers

**DOI:** 10.1007/s10147-016-1049-y

**Published:** 2016-10-11

**Authors:** Shingo Hatakeyama, Tohru Yoneyama, Yuki Tobisawa, Chikara Ohyama

**Affiliations:** Department of Urology, Hirosaki University Graduate School of Medicine, 5 Zaifu-chou, Hirosaki, 036-8562 Japan

**Keywords:** Prostate cancer, Biomarker, PSA, PHI, PCA3, S2,3PSA

## Abstract

The application of prostate-specific antigen (PSA) in prostate cancer (PC) screening, diagnosis, and prognosis has improved the clinical management of PC patients. However, the PSA assay has been faced with criticism due to its potential association with over-diagnosis and subsequent overtreatment of patients with indolent disease. The United States Preventive Services Task Force incited much debate over PSA-based screening in 2012 by recommending against this approach. However, the PSA assay remains the first-line tool for the early detection of PC. This debate highlights the unmet need for non-invasive PC biomarkers with greater sensitivity and specificity that are capable of distinguishing aggressive disease from indolent disease, predicting treatment response, and guiding treatment decisions. Recent investigations into putative PC biomarkers have focused on PSA isoform assays (prostate health index, 4-kallikurein panel), PC-associated genes in the urine (PCA3 and TMPRSS2-ERG), glycan-associated biomarkers (S2, 3PSA, GCNT1, and tri- and tetra-antennary serum *N*-glycans), and circulating tumor cells. Although substantial efforts to identify novel PC biomarkers that might replace PSA have been put forth, the majority of the putative PC biomarkers reported in the last few years are still under investigation or validation. This review provides an overview of the current state of PC biomarker research and focuses on a few promising PC biomarkers in development.

## Prostate-specific antigen (PSA) and the controversy in PSA screening

In 2015, prostate cancer (PC) was the most commonly diagnosed male malignancy, not only in western countries [1], but also in Japan [2]. PSA is the most commonly used biomarker for the early detection of PC. After the introduction of PSA testing, the rate of PC diagnosis increased and PC-associated mortality decreased. Elevated PSA levels are associated with an increased risk of PC, a higher pathological grade, and an increased risk of metastatic disease [3]. However, the use of PSA as a biomarker has a number of limitations. First, PSA is not a PC-specific biomarker. In addition, PSA levels are influenced by several factors, including age, acute prostatitis, ejaculation, catheterization, and certain medications. Furthermore, there is no precise value indicative of a lack of PC risk, and PSA levels cannot distinguish between indolent and aggressive disease, particularly at PSA levels below 20 ng/mL. In one study, the conventional cutoff value of 4 ng/mL PSA predicted PC in 10- or 12-core needle biopsies in only 30–40 % of patients [4]. In addition, ~15 % of men with serum PSA levels below 4 ng/mL are reported to be at risk for PC [5]. A precise PSA cut-off value that can facilitate the early detection of PC with high sensitivity and specificity in healthy men has not yet been defined. In addition, the ideal age at which to initiate or discontinue PSA testing, and the appropriate frequency of testing remain unclear. Two recent randomized trials evaluating the effect of PSA-based screening on mortality reduction reported conflicting results [6, 7]. Together, these findings prompted the United States Preventive Services Task Force to recommend against the use of PSA-based screening in 2012 [8]. Nevertheless, as there are no other reliable PC biomarkers to replace PSA, PSA screening remains the first-line assay for PC detection. As this approach is the subject of much debate and controversy, there is an unmet need for the identification of novel biomarkers with high sensitivity and specificity for detecting PC and predicting aggressive disease. Recently identified putative PC biomarkers are described in Table 1.

**Table 1:** Recently identified putative prostate cancer biomarkers

Biomarker	Biomaterial	Applications	Marker description
PHI	Serum	Diagnostic	Total PSA, [−2]proPSA, fPSA
PCA3	Urine	Diagnostic	PSA and PCA3 mRNA
4K score	Plasma	Diagnostic	Total PSA, fPSA, intact PSA
S2, 3PSA	Serum	Diagnostic	Aberrant glycosylation in serum PSA
TMPRSS2-ERG	Urine, blood tissue	Diagnostic	Fusion gene of ERG and transmembrane protease, serine 2
Mi-Prostate score	Urine	Diagnostic	PSA, PCA3 and TMPRSS2-ERG mRNAs
miRNA	Serum, plasma, urine	Diagnostic/aggressiveness	Altered miRNA expression profiles (miR-141, -375, -21, -107, 221, etc.)
Oncotype DX	Tissue	Aggressiveness	12 Cancer-related genes: androgen pathway (AZGP1, KLK2, SRD5A2, RAM13C), proliferation (TPX2), cellular organization (FLNC, GSN, TPM2, GSTM2) and stromal response (BGN, COL1A1 and SFRP4).
ProMark	Tissue	Aggressiveness	8 Proteins (DERL1, CUL2, SMAD4, PDSS2, HSPA9, FUS, phosphorylated S6, YBOX1)
Prolaris	Tissue	Aggressiveness	31 Cell cycle progression genes and 15 housekeeping genes in combination with PSA and Gleason score
Decipher GC	Tissue	Aggressiveness (metastasis)	22 RNAs form tissue after radical prostatectomy
GCNT1	Urine, tissue	Aggressiveness	Overexpression of the enzyme that forms core 2-branched *O*-glycans
*N*-glycans	Serum	Aggressiveness	Aberrant glycosylation in serum *N*-glycans
AR-V7	Blood	Aggressiveness	AR-V7 expression in CTCs

## PSA isoforms

**Fig. 1:**
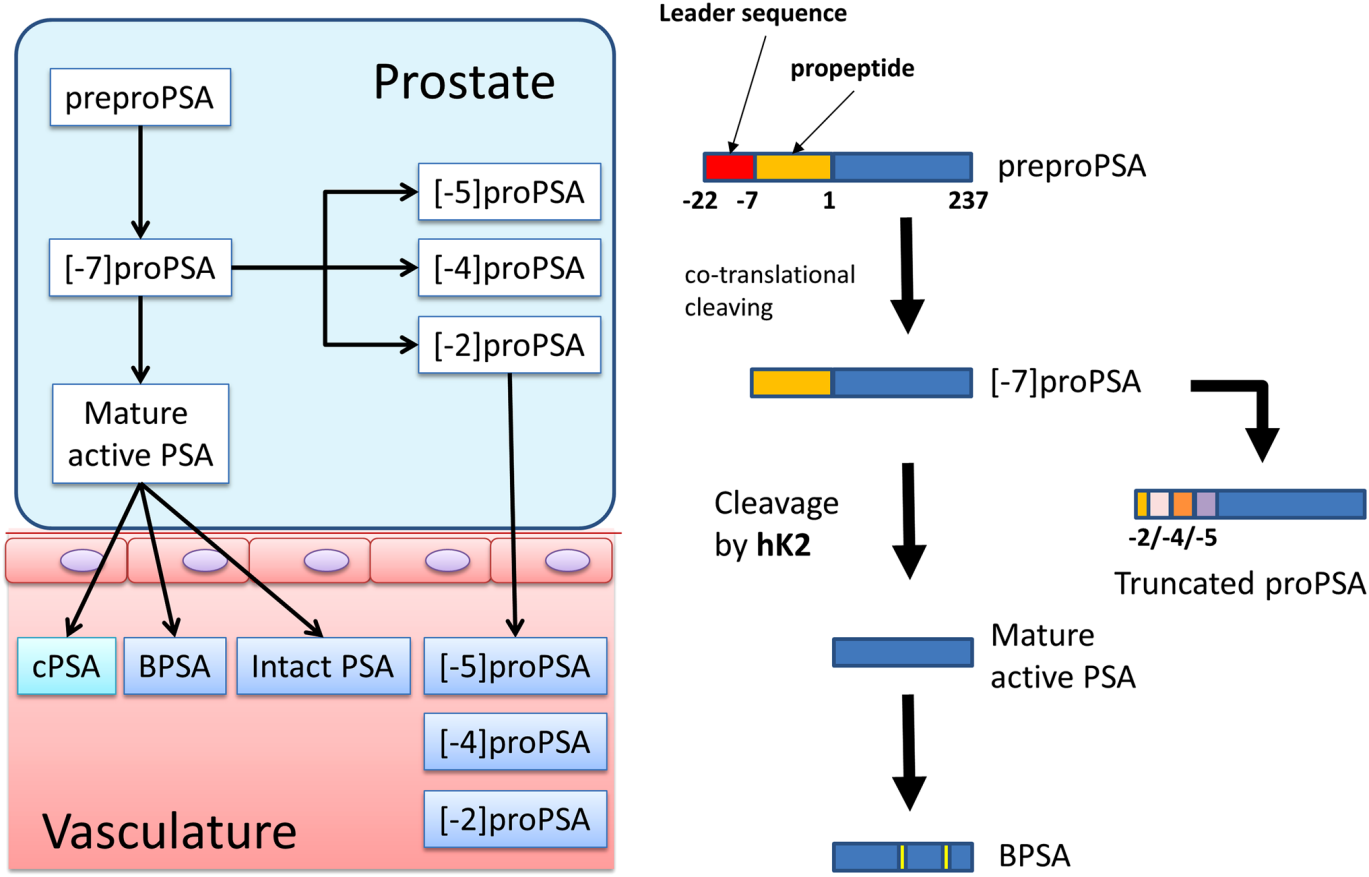
PSA synthesis and PSA isoforms. PSA synthesis begins with the cleavage of the proenzyme PSA (proPSA) leader sequence from preproPSA. There are several truncated isoforms of proPSA, including [−7]proPSA, [−5]proPSA, and [−2]proPSA. [−2]proPSA is the predominant proPSA isoform in tumor extracts, indicating that it has the potential to play a role in the early detection of PC and the prediction of aggressive disease. The cleavage of the propeptide in proPSA by human kallikrein 2 (hk2) generates the mature PSA molecule. Benign PSA (BPSA), intact PSA, and [−2]proPSA exist as free PSA molecules in the serum. *cPSA* complexed PSA

Serum PSA predominantly exists in a complex with α-1-antichymotrypsin. Levels of unbound PSA, referred to as free PSA (fPSA), are calculated using the following formula fPSA = total PSA − α-1-antichymotrypsin-bound PSA. %fPSA is associated with a greater specificity for detecting PC in men with a total PSA between 4 and 10 ng/mL [9, 10]. Three isoforms of fPSA are found in the serum: (1) proPSA, (2) intact PSA, and (3) benign PSA. There are also several truncated isoforms of proPSA, including [−2]proPSA, [−5]proPSA, and [−7]proPSA. [−2]proPSA is the predominant proPSA isoform in tumor extracts [11], suggesting that it has the potential to play a role in the early detection of PC and the prediction of aggressive disease [12] (Fig. 1).

### Prostate health index (PHI)

The prostate health index (PHI) is an assessment of the three PSA isoforms. PHI is calculated using the following formula: ([−2]proPSA/fPSA) × PSA^1/2^. The test was developed by Beckman Coulter in partnership with the NCI Early Detection Research Network. The aim of PHI is to distinguish between malignant and benign prostate conditions in men 50 years or older with normal digital rectal exam (DRE) results and PSA levels of 4–10 ng/mL, and to ultimately reduce the number of unnecessary biopsies performed. Several studies have suggested that PHI significantly improves prostate cancer detection in high-risk cases [11, 13] and that is associated with PC aggressiveness [12]. PHI and [−2]proPSA were approved by the US Food and Drug Administration (FDA) in 2012. PHI has been gaining acceptance worldwide and has been approved for clinical use in more than 50 countries. However, it is not yet recommended as a first-line screening method for all patients due to a lack of sufficient data. Further prospective analysis of this approach is needed to support its use as a first-line screening tool for PC in all patients.

### 4K score

The 4K score is an assessment of kallikrein-related peptide 2 (hK2) and the three PSA isoforms included in the PHI (total PSA, fPSA, and intact PSA). The 4K score also incorporates clinical information, including age and history of prior negative biopsy, to provide an estimate of the probability of biopsy-confirmed PC. Retrospective studies reported that the 4K score was more accurate in predicting clinically diagnosed PC [14] and aggressive disease [15] compared with PSA and age. A recent meta-analysis reported that the 4K score is associated with a improvement of 8–10 % in predicting biopsy-confirmed PC, indicating that the use of the 4K score could potentially eliminate the number of prostate biopsies currently conducted by an estimated 48–56 % [16]. Although the accuracy of the 4K score highlights the drawbacks of PSA screening, it is not yet FDA-approved or included in current guideline recommendations. Furthermore, as no comparative study has been reported, it is unclear if the predictive accuracy of the PHI and the 4K score differs. Therefore, additional prospective studies are needed to evaluate their use as screening tools for the early detection of PC.

## Genomic biomarkers in urine

### Progensa PCA3 assay

Prostate cancer gene 3 (PCA3) is prostate-specific noncoding mRNA that is strongly expressed in patients with PC. The PCA3 assay measures the concentration of PCA3 and PSA RNA molecules. The PCA3 score is calculated as the ratio of PCA3 RNA molecules to PSA RNA molecules (PCA3 score) in post-DRE urine specimens. A PCA3 score less than 25 indicates a low risk of PC [17]. The test was approved by the FDA in 2012. It is used to help determine if a repeat biopsy is necessary for men with a previous negative biopsy. A meta-analysis of 11 clinical studies reported that the sensitivity of the PCA3 assay ranged from 53 to 69 % and the specificity ranged from 71 to 83 % [18]. A more recent meta-analysis of 11 studies reported that a sensitivity and specificity of 72 and 53 %, respectively, was associated with a PCA3 score cut-off of 20 [19]. These findings indicate that the PCA3 test might be more accurate compared with other methods used for the early detection of PC. The Progensa PCA3 assay has been included in the EAU guidelines for repeat biopsy decision-making.

### The TMPRSS2-ERG fusion gene

Gene rearrangements have been observed in multiple cancers, and they are especially prevalent in leukemia (e.g., the Philadelphia chromosome). The TMPRSS2-ERG fusion gene, comprising the androgen-responsive genes transmembrane protease, serine 2 (TMPRSS2), and estrogen-regulated gene (ERG), was observed in ~40–80 % of prostate cancers in 2005 [20]. Both genes are located on chromosome 21. The TMPRSS2-ERG score is calculated using the following formula: (TMPRSS2-ERG mRNA/PSA RNA copies) × 100,000. Levels of urine TMPRSS2-ERG appear to be associated with clinically significant PC [21]; however, the prognostic significance of TMPRSS2-ERG is unclear. A recent meta-analysis suggested that TMPRSS2-ERG overexpression is associated with tumor stage, but that it is not associated with disease recurrence or mortality in men treated with radical prostatectomy (RP) [22].

### Mi-Prostate score (MiPS)

TMPRSS2-ERG is a PC-specific fusion gene. However, most prostate tumors have multiple foci and the expression of TMPRSS2-ERG is thought to be heterogeneous. The MiPS overcomes this limitation by assessing multiple PC-associated parameters. MiPS combines the prognostic significance of urine TMPRSS2-ERG and PCA3 with serum PSA to generate a PC risk assessment score. In a recent validation study with 1225 patients, MiPS was superior to serum PSA alone in predicting biopsy-confirmed PC and high-grade PC [23]. However, this test is not yet FDA-approved.

## Genomic and protein biomarkers in tissue

### Oncotype DX test

The Oncotype DX test is a multi-gene expression assay developed for formalin-fixed paraffin-embedded diagnostic prostate needle biopsies. The assay measures the expression of 12 cancer-related genes representing 4 distinct biological functions: androgen signaling (*AZGP1, KLK2, SRD5A2,* and *RAM13C*), proliferation (*TPX2*), cytoskeletal organization (*FLNC, GSN, TPM2, GSTM2*), and the stromal response (*BGN, COL1A1*, and *SFRP4*). Five reference genes have been included to normalize the data and control for variability. The gene expression levels are algorithmically combined to calculate a genomic prostate score (GPS). The Oncotype DX test has been validated as a predictor of prostate cancer aggressiveness, and it facilitates prostate cancer risk stratification to help guide treatment decision-making [24]. It is included as a potential option in the National Comprehensive Cancer Network (NCCN) guidelines of 2015, with the disclaimer that further studies of the assay are still needed [25].

### Prolaris test

The Prolaris test is a multi-gene expression assay (46 genes) developed for formalin-fixed paraffin-embedded tissue derived from prostate needle biopsies. It is used in conjunction with the Gleason score and serum PSA to predict prostate cancer aggressiveness. The Prolaris test assesses disease progression by quantitatively analyzing the expression of 31 genes associated with cell cycle progression and 15 housekeeping genes. Low expression levels of these genes are associated with a low risk of disease progression, whereas high expression levels are indicative of a higher risk of disease progression [26]. The Prolaris test is included in the NCCN 2015 guidelines.

### Decipher genomic classifier (Decipher GC)

Decipher is a genomic classifier (GC) test that uses a whole-transcriptome microarray assay to analyze the expression of 22 genes in formalin-fixed paraffin-embedded prostate cancer specimens obtained from RP. Decipher GC can predict the risk of metastasis following RP. In a clinical validation study, Decipher was a more accurate predictor of metastasis post-RP compared with individual clinical variables, with an area under the curve (AUC) of 0.79 for predicting 5-year metastasis risk [27]. A genomic risk stratification assay using the primary tumor can identify patients at a high risk for metastasis and potentially lethal prostate cancer, thereby providing information that can improve treatment decision-making post-RP. The Decipher score is included in the NCCN 2015 guidelines as a post-RP prognostic marker. The specific genes evaluated and the formula used to calculate the Decipher GC score have not been published.

### ProMark

ProMark is a prognostic assay that analyzes the expression of 8 protein biomarkers in formalin-fixed paraffin-embedded tissue obtained from prostate needle biopsies. It is used to predict prostate cancer aggressiveness, particularly in patients with a Gleason score 3 + 3 or 3 + 4. ProMark quantitatively analyzes the levels of 8 proteins (DERL1, CUL2, SMAD4, PDSS2, HSPA9, FUS, phosphorylated S6, and YBOX1) in biopsy tissue sections using an automated immunofluorescence method. The protein levels are used to calculate a risk score that has been clinically validated as an independent predictor of prostate cancer aggressiveness [28]. The ProMark score can help distinguish patients that should be actively monitored from those that require therapeutic intervention.

## Cancer-associated glycan biomarkers

Cancer-associated glycan aberrations are frequently observed in tumors. The majority of tumor markers, including PSA, are glycoproteins that have glycosylation sites in their amino acid sequence. Importantly, each glycan exhibits specific cancer-associated carbohydrate aberrations compared with its normal counterpart, and these aberrations can be detected using specific monoclonal antibodies or lectin.

### Aberrant serum PSA glycosylation (S2,3PSA)

**Fig. 2:**
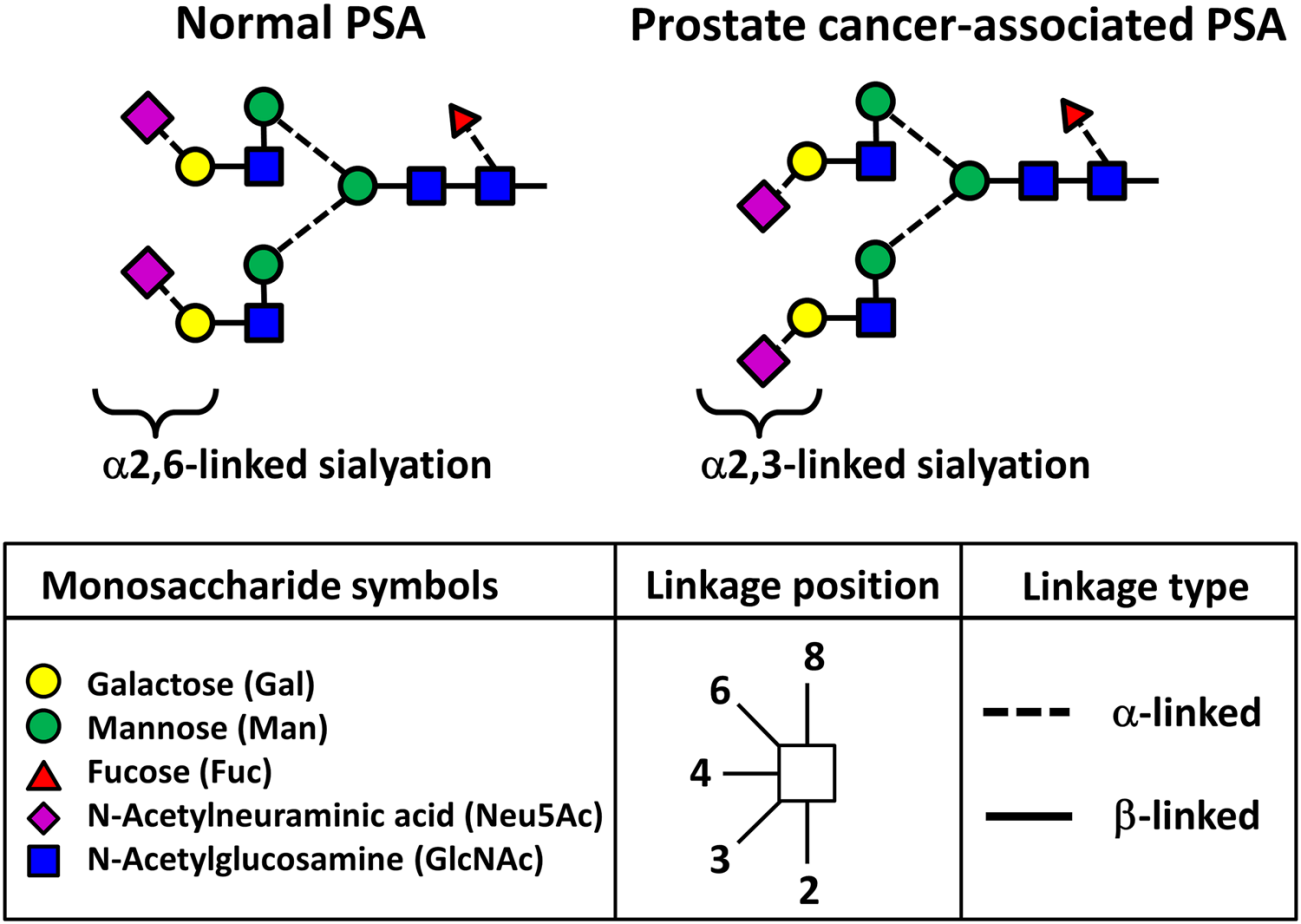
Aberrant glycosylation of PSA *N*-glycans (S2, 3PSA) in PC. In healthy patients, the terminal sialic acid of PSA is predominantly α2,6-linked to galactose residues. In patients with PC, the terminal sialic acid is predominantly α2,3-linked to galactose residues

PSA is a glycoprotein with a single *N*-glycosylation site at an asparagine (*N*) residue 45 amino acids from the N-terminus. In patients with PC, the terminal *N*-glycan structure of PSA is rich in sialic acid α2,3-linked galactose, whereas the terminal *N*-glycan structures of PSA from healthy patients are predominantly α2,6-linked [29] (Fig. 2). Based on this finding, Yoneyama et al. successfully developed a novel assay using a magnetic microbead-based immunoassay to detect α2,3-linked sialylation on free PSA (S2, 3PSA) [30]. The diagnostic accuracy of S2, 3PSA was associated with an AUC of 0.84, and the sensitivity and specificity of the assay was 95.0 and 72.0 %, respectively, a significant increase compared with PSA or %fPSA. Although the study was small and preliminary, the results suggest that assays measuring cancer-associated glycan alterations in serum S2, 3PSA might improve the accuracy of early PC detection and reduce unnecessary prostate biopsies.

### Highly branched (tri- or tetra-antennary) serum *N*-glycans

Although cancer-associated glycan alterations represent potential cancer biomarkers, glycan analysis has not been incorporated into clinical use because the protocols for preparing glycan derivatives vary depending on the analytical method and conducting these protocols requires specialized expertise. Therefore, a practical procedure for analyzing a large number of glycan samples in biological materials such as serum is not currently available.

Recently, an approach that combines high-throughput, quantitative *N*-glycomics with mass spectrometry analysis was developed. The technique is based on a chemoselective glycan enrichment technology that enables the purification of oligosaccharides from 10 μL of crude glycoproteins. Preliminary results indicate that serum *N*-glycan analysis is a promising approach to screening diagnostic and prognostic markers associated with multiple types of cancer [31, 32]. Ishibashi et al. evaluated the potential predictive value of serum *N*-glycomics in patients with castration-resistant PC (CRPC) [33]. They used serum *N*-glycomics with the glycoblotting method to analyze 80 healthy volunteers, 286 patients with benign prostatic hyperplasia, 258 patients with early-stage PC, 46 patients with PC that had been treated with androgen deprivation therapy (ADT), and 68 patients with CRPC. They found that tri- and tetra-antennary *N*-glycans were significantly enriched in patients with CRPC compared with the other groups. The longitudinal follow-up of highly branched *N*-glycan levels predicted CRPC despite castrate levels of testosterone. These results suggest that the overexpression of specific *N*-glycans might be associated with CRPC and that it might represent a predictive CRPC biomarker.

### Core2 β-1,6-*N*-acetylglucosaminyltransferase (GCNT1)

Core2 β-1,6-*N*-acetylglucosaminyltransferase-1 (GCNT1) is an enzyme that plays a key role in the formation of core 2-branched *O*-glycans, and GCNT1 expression is associated with the progression of several types of cancer [34–37]. GCNT1 expression is strongly associated with disease aggressiveness in patients with PC [38–40]. Kojima et al. recently evaluated the utility of GCNT1 for the detection of aggressive PC using immunohistochemistry and immunoblotting assays in post-DRE urine [41]. They reported that over 90 % of GCNT1-positive PC patients with high concentrations of PSA presented with extracapsular extensions, further confirming that GCNT1 expression is strongly associated with aggressive PC. Further research is needed to develop an efficient assay that can detect GCNT1 in post-DRE urine and facilitate the use of GCNT1 as a marker of PC aggressiveness in the clinical setting.

## Circulating tumor cells (CTCs)

Circulating tumor cells (CTCs) are tumor cells that have shed into the peripheral circulation from solid malignancies. CTCs have generally been considered surrogates for metastatic cells, and CTCs levels in patients undergoing treatment have proven to be a response marker with a strong independent prognostic value [42]. In addition, CTCs have been characterized as a ‘‘liquid biopsy” that can facilitate the real-time monitoring of therapeutic efficacy, and the identification of therapeutic targets and resistance mechanisms [43].

Recently, the presence of the AR splice variant 7 (AR-V7) in CTCs was shown to be strongly associated with resistance to anti-AR therapy [44]. The presence of AR-V7 in CTCs was associated with poor overall survival, with a hazard ratio (HR) of 6.9 [95 % confidence interval (CI) 1.7–28.1, *P* = 0.002] in patients receiving enzalutamide cohort and an HR of 12.7 (95 % CI 1.3–125.3, *P* = 0.006) in patients receiving abiraterone cohort. The same group also reported that the presence of AR-V7 did not significantly influence the effect of taxane treatment [45]. More recently, Scher et al. reported similar results from a prospective study of 191 blood samples from patients with metastatic CRPC. Although the proportion of patients with AR-V7-positive CTCs in this study was relatively low (34/191 samples, 18 %), AR-V7 positive patients were resistant to AR inhibition therapy, were on therapy for less time, and experienced a reduction in radiographic progression-free survival and overall survival compared with patients with AR-V7-negative CTCs [46]. However, there are substantial limitations to the clinical application of AR-V7 monitoring as only half of patients have detectable CTCs, CTSs cannot be stored, and the procedure is costly and can only be conducted in specialized labs. Although AR-V7 appears to be a promising prognostic factor, additional studies and technological innovations are needed to facilitate its widespread clinical use.

## Conclusion and perspectives

**Fig. 3:**
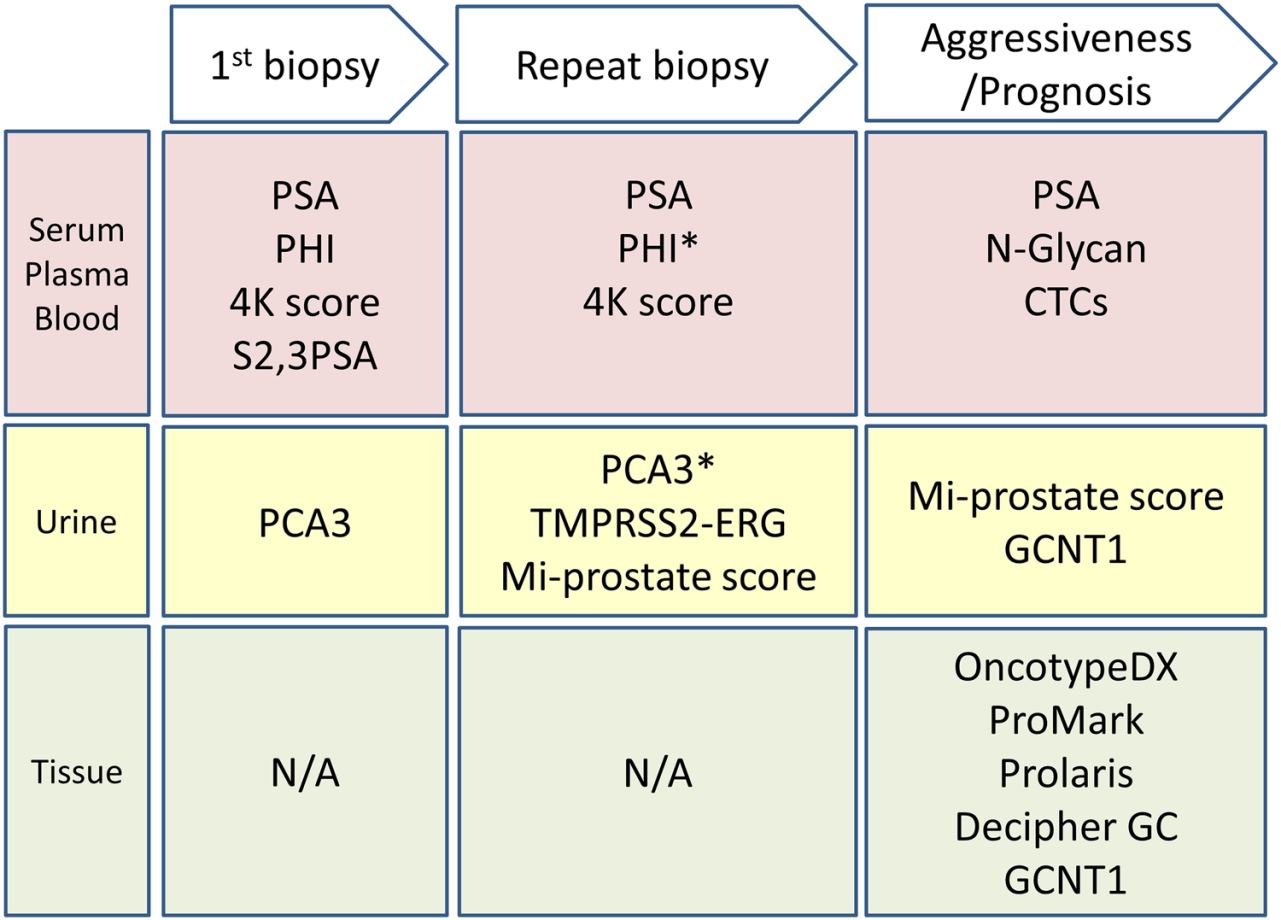
Overview of potential prostate cancer biomarkers. Novel biomarkers were classified according to screening to prognostic phase based on types of biomaterials. *Asterisk* US FDA approved, *N/A* not available

Although serum PSA has been used as a prostate cancer biomarker for several decades, studies demonstrating its limitations have incited much controversy and debate. The ideal PC biomarker would be capable of distinguishing PC from benign prostate conditions and differentiating between aggressive and indolent tumors. Recent technological innovations have led to substantial improvements in biomarker screening assays. An overview of the emerging prostate cancer biomarkers described in this review are summarized in Fig. 3. Specifically, two new tests designed to help determine the need for prostate biopsy have recently been approved by the US FDA. Currently, the incorporation of new biomarkers appears to be a promising approach for improving the sensitivity and specificity of PSA assays. However, comparative studies evaluating these various biomarker assays are still needed, as are studies with larger sample sizes and improvements in cost effectiveness. Large-scale, prospective trials can help evaluate the utility of these new approaches in multiple clinical contexts. The use of novel biomarkers that can serve as alternatives to PSA appears to be a promising approach to improving risk assessment strategies and has the potential to improve outcomes in patients with PC.
